# The Comparison of the Results of Microfracture and Mosaicplasty in Talus Osteochondral Lesions

**DOI:** 10.7759/cureus.61217

**Published:** 2024-05-28

**Authors:** Namık Kılınçcıoğlu, Aydıner Kalacı

**Affiliations:** 1 Orthopaedics and Traumatology Department, Osmaniye State Hospital, Osmaniye, TUR; 2 Orthopedics and Traumatology Department, Tayfur Ata Sokmen Faculty of Medicine, Mustafa Kemal University, Hatay, TUR

**Keywords:** cartilage defect, talus, microfracture, mosaicplasty, osteochondral lesion

## Abstract

Purpose: This study aims to compare the mid-term functional outcomes of microfracture and mosaicplasty techniques in talus osteochondral lesions.

Materials and methods: This study consists of 47 patients with talus osteochondral lesions who underwent arthroscopic surgery. These patients were divided into two groups: microfracture (28 patients) and mosaicplasty (19 patients). The American Orthopedic Foot and Ankle Society (AOFAS) scoring system was used to evaluate ankle function, and the Visual Analog Scale (VAS) score was used for pain assessment.

Results: The mean follow-up period was 26 months (range 10-36 months). It was determined that the mean preoperative AOFAS score of individuals in the mosaicplasty group was 38.84±2.83, and the postoperative AOFAS score was 78.79±3.91. A statistically significant difference was found between the two measurements of AOFAS scores (preoperative and postoperative) in the mosaicplasty group (*t=33.756; p<0.001). The effect size for this difference observed in the mosaicplasty group was determined to be r=0.992 (large). Similarly, a statistically significant difference was found between the two measurements of AOFAS scores (preoperative and postoperative) in the microfracture group (*t=28.152; p<0.001). The effect size for this difference observed in the microfracture group was determined to be r=0.983 (large).

Conclusion: We believe that both treatment methods have similar positive effects on pain and ankle function. However, larger controlled studies with longer follow-up periods are needed to reach a definitive conclusion.

## Introduction

Osteochondral lesions of the talus are caused by the isolated separation of the talus articular cartilage, either along with or without the subchondral bone [1]. While these lesions are most commonly seen in the knee joint, they occur second most frequently in the ankle joint. Although their etiology has not been fully elucidated, trauma is the most significant etiological factor. The role of trauma in lateral lesions is more common compared to medial lesions [2].

Early diagnosis and treatment of these types of joint cartilage lesions are of great importance for preserving joint function, relieving pain, and preventing osteoarthritis [3]. Both conservative and surgical treatment options are available. Physical therapy applications and oral or intra-articular medications are conservative treatment methods, while surgical treatment methods include debridement, bone marrow stimulation (microfracture, drilling), cell-free scaffold implants, autologous chondrocyte implantation, matrix-associated autologous chondrocyte implantation, autologous osteochondral transplantation, mosaicplasty, and allograft transplantation [4,5]. Currently, the preferred initial treatment for talus OCD (osteochondral defect) surgery is arthroscopic debridement combined with excision and microfracture [6]. On the other hand, mosaicplasty provides high durability depending on the quality of the transferred hyaline cartilage and is particularly preferred in young patients with high expectations [7]. The study aims to compare the effectiveness of mosaicplasty and microfracture methods in the mid-term on pain and ankle function in talus osteochondral defects.

## Materials and methods

This study consists of 47 patients followed at our hospital between 2008 and 2020 due to talus osteochondral lesions. Ethics committee approval for the study was received from the Hatay Mustafa Kemal University Faculty of Medicine Ethics Committee on May 12, 2022, with decision number 23. Informed consent was obtained from all patients participating in the study. Diabetic, morbidly obese patients with ankle arthrosis and those over 65 years of age were excluded from the study. Of the patients, 26 (55.3%) were female and 21 (44.7%) were male, with a mean age of 39.02±14.71 years. Patients were divided into two groups: the microfracture group (28 patients, 59.6%) and the mosaicplasty group (19 patients, 40.4%). All microfracture procedures were performed arthroscopically. However, a mosaicplasty procedure was performed after diagnostic arthroscopy with a medial malleolar osteotomy. Lesions were classified according to the Hepple classification. A mosaicplasty called for follow-up visits on the 11th day, 6th week, 6th month, and 1st year after the procedure. The procedure was performed on patients with lesions larger than 1.5 cm2 in width. The mean follow-up period was 26 months. Patients were called for follow-up visits on the 11th day, 6th week, 6th month, and 1st year after the procedure.

Surgical technique

Microfracture

Standard anterior portals were used for arthroscopic entry. The osteochondral fragment was removed and excised. Necrotic and granulation tissue were curetted until reaching the underlying live, hard bone tissue. Then, microfracture was performed by making 3-4 holes per cm^2^ with 20°, 40°, and 90° awls. Observation showed fat particles coming out of the holes. The tourniquet was released after the procedure, and bleeding from the holes was observed. No drains were placed in the ankle joints.

Mosaicplasty

**Figure 1:**
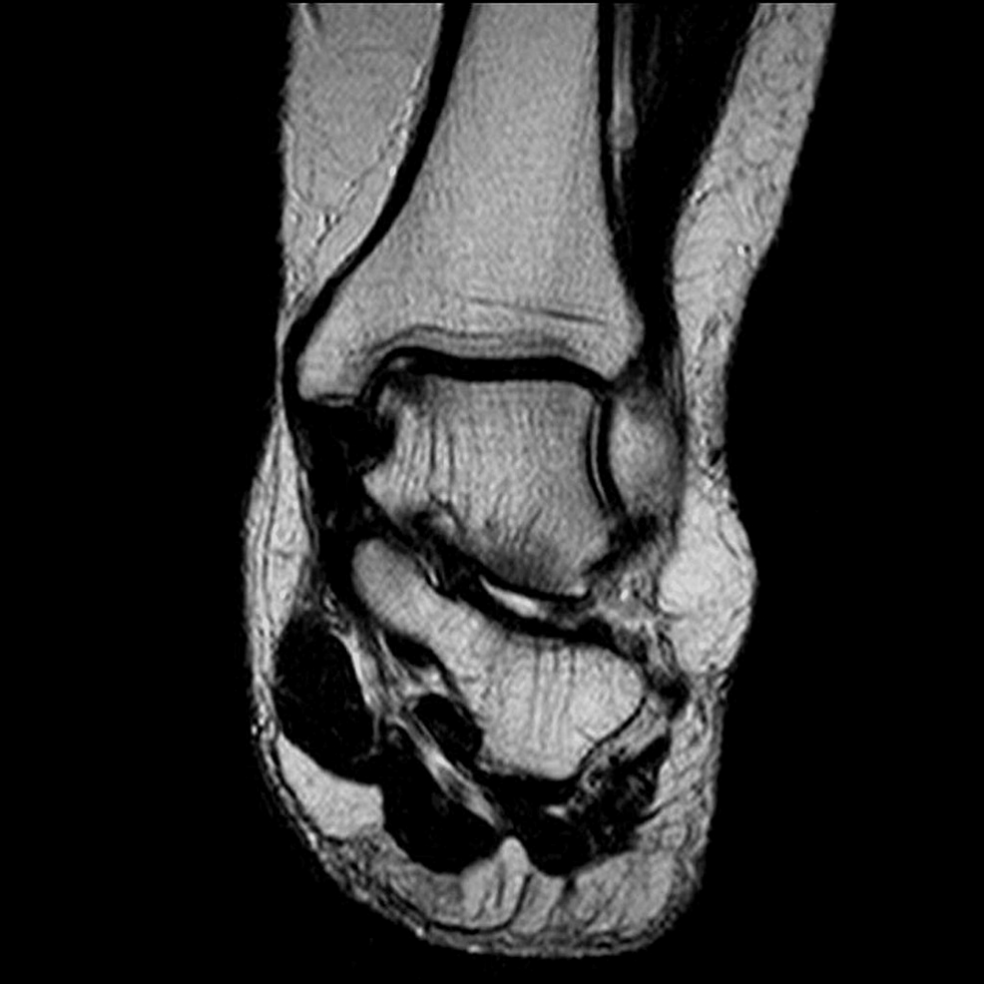
Talus medial osteochondral lesion. Preoperative MRI images: coronal section

**Figure 2:**
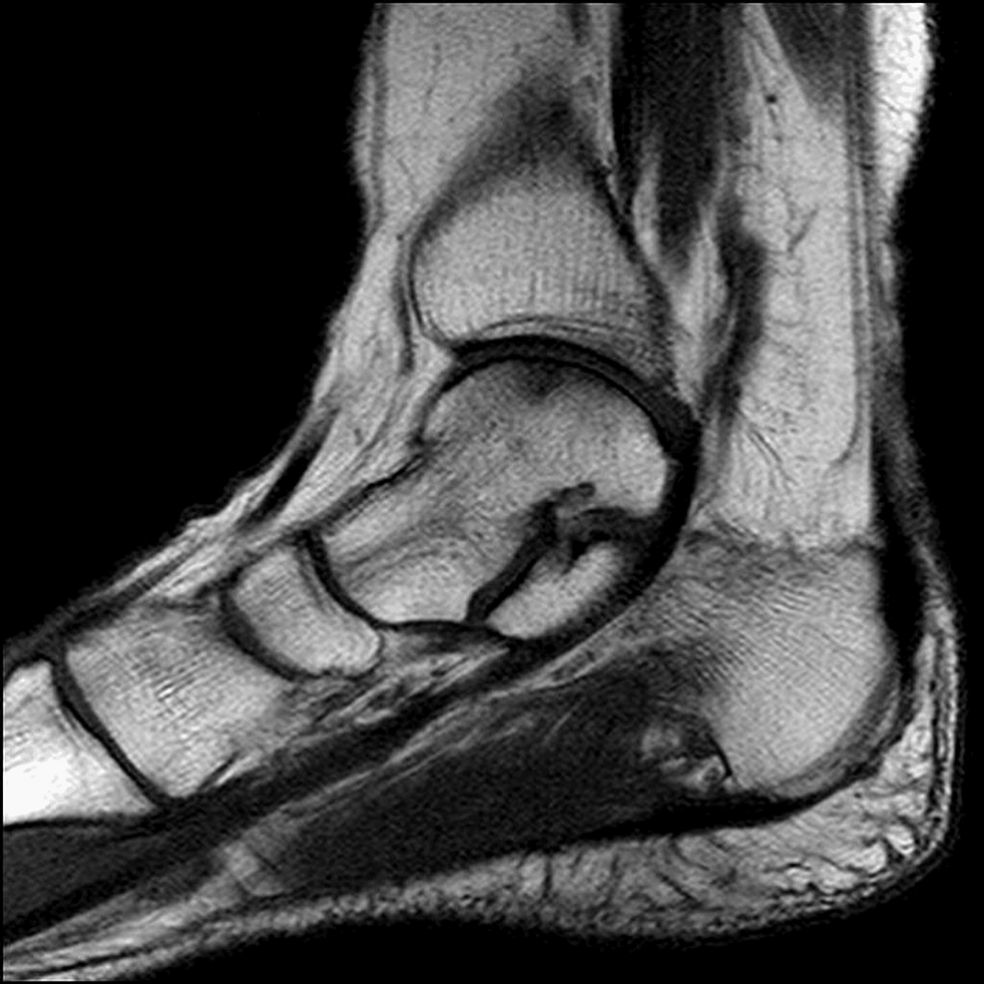
Talus medial osteochondral lesion. Preoperative MRI images: axial section

**Figure 3:**
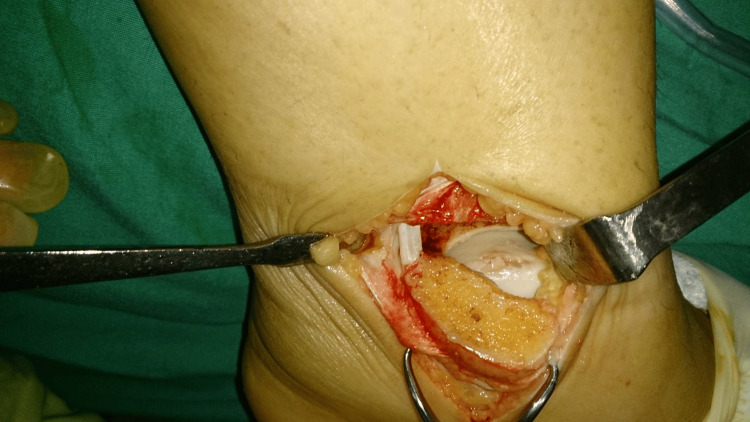
An intraoperative view of the articular surface of the medial talar dome with an osteochondral lesion exposed through a medial malleolar osteotomy

**Figure 4:**
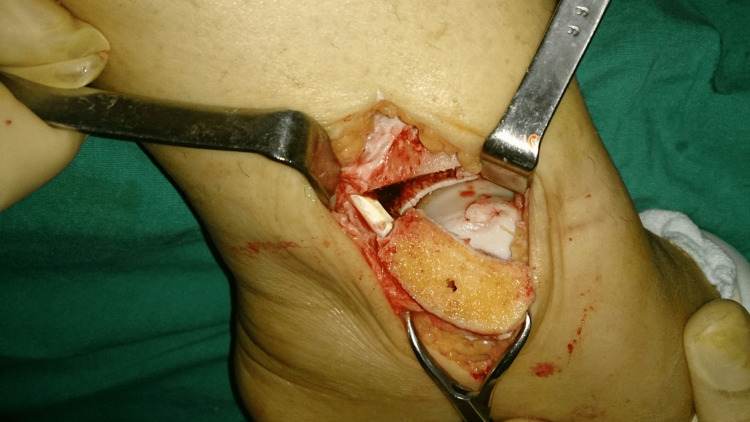
An intraoperative view of the talar dome after reconstruction of a medial defect using an autologous osteochondral graft

Before open surgery, ankle arthroscopy was performed to better evaluate the cartilage lesion. Through lateral knee arthrotomy, grafts were harvested from the non-weight-bearing surface of the lateral femoral condyle using the osteochondral autograft transfer system set. Depending on the size and depth of the lesion, we collected grafts with a diameter of 6, 8, or 10 mm and a depth of 12-15 mm. Access to the talus osteochondral lesion was achieved through a medial malleolar osteotomy. The lesion was then excised, and the space was curetted and debrided. A socket of appropriate width and depth was created for the graft. The cylindrical graft was placed in the socket, and the osteotomy line was reduced and fixed with cannulated screws. The incision was closed, and a short leg cast was applied (Figures 1-4).

Statistical analysis

The normal distribution of continuous variables in the study was evaluated graphically and with the Shapiro-Wilks test. Descriptive statistics were presented using Mean±Standard Deviation and Median (Minimum-Maximum) values. The Mann-Whitney U test was used to compare age, mean follow-up duration, preoperative VAS, and postoperative VAS values between the mosaicplasty and microfracture groups. An independent sample t-test was used to compare preoperative AOFAS and postoperative AOFAS values between the mosaicplasty and microfracture groups. A paired samples t-test was used to examine whether there were differences in AOFAS values at measurement times (preoperative-postoperative), and a Wilcoxon signed-rank test was used to examine whether there were differences in VAS values at measurement times (preoperative-postoperative). Cross-tables were created to compare categorical variables between the Mosaicplasty and Microfracture groups, and the number (n), percentage (%), and chi-square (χ^2) test statistics were provided. Effect size (r) was calculated for observed changes in each group, and if the r value was between 0.10-0.29, it was interpreted as a small effect size, if it was between 0.30-0.49, it was interpreted as a medium effect size, and if it was ≥0.50, it was interpreted as a "large" effect size. IBM Corp. Released 2012. IBM SPSS Statistics for Windows, Version 21.0. Armonk, NY: IBM Corp. and MS-Excel 2007 programs were used for statistical analysis and calculations. A statistical significance level of p<0.05 was accepted.

## Results

The characteristics of the groups are presented in Table 1. It was found that individuals' age, gender, lesion side, history of trauma, and mean follow-up duration were similar between the groups according to the method (p>0.05). A statistically significant difference was observed in terms of stage distribution according to the method (χ^2=13.427, p=0.004). Among individuals who underwent mosaicplasty, 10.5% (n=2) were in stage 2, 26.3% (n=5) were in stage 3, 36.9% (n=7) were in stage 4, and 26.3% (n=5) were in stage 5, while among those who underwent microfracture, 21.4% (n=6) were in stage 2, 57.2% (n=16) were in stage 3, and 21.4% (n=6) were in stage 4.

**Table 1:** Comparison of demographic and clinical characteristics in mosaicplasty and microfracture groups z: Mann-Whitney U Test, X2: chi-square test, *Fisher Exact test results, n: number of cases, p<0.05: significant, SD: standard deviation, %: percent

	METHOD				
	All cases (n=47)	Mosaicplasty (n=19)	Micro fracture (n=28)	Test statistics	
	Average± SD Median (Min-Max)	Average± SD Median (Min-Max)	Average± SD Median (Min-Max)	z; c^2^	p-value
Age (years)	39.02±14.71	44.26±15.22	35.46±13.48	z=1.911	0.056
	40.0 (15-63)	48.0 (15-61)	35.0 (15-63)		
gender, n (%)					
female	26 (55.3)	12 (63.2)	14 (50.0)	χ2=0.793	0.373
male	21 (44.7)	7 (36.8)	14 (50.0)		
Lesion side, n (%)					
right	21 (45.7)	10 (52.6)	11 (40.7)	χ2=0.636	0.425
left	25 (54.3)	9 (47.4)	16 (59.3)		
Trauma, n (%)					
no	8 (17.0)	3 (15.8)	5 (17.9)	-	0.589*
yes	39 (83.0)	16 (84.2)	23 (82.1)		
stage, n (%)					
stage-2	8 (17.0)	2 (10.5)	6 (21.4)	χ2=13.427	0.004
stage-3	21 (44.7)	5 (26.3)	16 (57.2)		
stage-4	13 (27.7)	7 (36.9)	6 (21.4)		
stage-5	5 (10.6)	5 (26.3)	0 (0.0)		
Median duration of follow up (months)	26.62±4.18	26.26±4.27	26.86±4.19	z=0.479	0.632
	26.0 (19-36)	26.0 (19-36)	25.5 (21-36)		

In the mosaicplasty group, the preoperative AOFAS mean was 38.84±2.83, while in the microfracture group, it was 45.29±4.74. A statistically significant difference was found between the mosaicplasty and microfracture groups in terms of preoperative AOFAS values (t=5.309, p<0.001). Statistically significant differences were also observed in the AOFAS values over time (Preop, Postop) within both the mosaicplasty (*t=33.756; p<0.001) and microfracture (*t=28.152; p<0.001) groups. The effect size for this difference in the mosaicplasty group was determined to be r=0.992 (large), while in the microfracture group, it was r=0.983 (large). However, there was no statistically significant difference between the groups in terms of postoperative AOFAS values (t=1.148, p=0.257) (Table 2).

**Table 2:** Comparison of Aofas values in mosaicplasty and microfracture groups t: independent sample t test, *t: dependent sample t test, r: effect size, SD: standard deviation, Aofas: American Orthopaedic Foot & Ankle Society, p<0.05: significant, n: number of cases

	Mosaicplasty (n=19)		Microfracture (n=28)			
	Average± SD	Median (Min-Max)	Average± SD	Median (Min-Max)	p (Group)	
Aofas						
Preop	38.84±2.83	39.0 (34-43)	45.29±4.74	45.0 (33-56)	t=5.309	p<0.001
Postop	78.79±3.91	79.0 (71-85)	77.32±4.55	77.0 (68-88)	t=1.148	p=0.257
p(time)	*t=33.756; p<0.001		*t=28.152; p<0.001			
r	0.992		0.983			

In the mosaicplasty group, the preoperative VAS mean was 8.11±0.81, while in the microfracture group, it was 7.11±0.96. A statistically significant difference was found between the mosaicplasty and microfracture groups in terms of preoperative VAS values (z=3.253, p=0.001). Statistically significant differences were also observed in the VAS values over time (preoperative and postoperative) within both the mosaicplasty (*z=3.842; p<0.001) and microfracture (*z=4.697; p<0.001) groups. The effect size for this difference in the mosaicplasty group was determined to be r=0.671 (large), while in the microfracture group, it was r=0.670 (large). However, there was no statistically significant difference between the groups in terms of postoperative VAS values (z=1.550, p=0.121) (Table 3).

**Table 3:** Comparison of VAS values in mosaicoplasty and microfracture groups z: Mann-Whitney U Test, *z: Wilcoxon signed rank test, r: effect size, n: number of cases, p<0.05: significant, SD: standard deviation, VAS: Visual analogue scale

	Mosaicplasty (n=19)		Microfracture (n=28)			
	Average± SD	Median (Min-Maks)	Average± SD	Median (Min-Maks)	p (Group)	
VAS						
Preop	8.11±0.81	8.0 (7-9)	7.11±0.96	7.0 (6-9)	z=3.253	p=0.001
Postop	3.11±0.99	3.0 (2-5)	3.50±0.79	3.0 (2-5)	z=1.550	p=0.121
P (time)	*z=3.842; p<0.001		*z=4.697; p<0.001			
r	0.671		0.670			

In our cases, we did not encounter any complications requiring reoperation. However, we experienced knee pain in five (10.6%) cases in the mosaicplasty group.

## Discussion

The most significant finding in this study is that microfracture and mosaicplasty techniques have similar effects on pain and ankle function in talus osteochondral lesions.

Curettage and drilling, along with arthroscopic debridement, are considered the gold standard treatments for small lesions. Satisfactory results with this technique have been reported to be as high as 85-87% [8,9]. However, this technique has resulted in fibrocartilaginous tissue filling the defect and has shown suboptimal outcomes for larger lesions, cystic lesions, and lesions requiring revision surgery [10-12]. This has led to the development of surgical techniques aiming to restore the superior biomechanical properties of hyaline cartilage, including osteochondral transplantation (OATS), mosaicplasty, osteochondral allograft, and autologous chondrocyte implantation (ACI) [13-22].

We can repair small articular cartilage defects using the mosaicplasty method, but there are still some unresolved issues, such as donor site morbidity [23]. It has been reported in the literature that donor site morbidity could be up to 15% [24]. Al-Shaikh et al. treated 19 talus OCD patients with the mosaicplasty method and reported only mild knee pain in two patients (10.5%) [13]. Gautier et al., in a case series of 11 patients, reported knee pain in only one patient (9%) [25]. Similarly, in our series of 47 cases, we experienced knee pain in a similar proportion (five patients, 10.6%), as reported in the literature.

Valderranbo found good to excellent results in 92% of his patients treated with mosaicplasty for talar osteochondral lesions. However, he emphasized the need for caution regarding donor site morbidities and their potential association with the development of patellofemoral osteoarthritis later in life [26]. We did not encounter patellofemoral pathology in mosaicplasty cases. This could be attributed to our relatively short follow-up period (average of 26 months). We believe that a longer follow-up period is needed to reach a definitive conclusion on this matter.

On the other hand, Baltzer and Arnold noted a 5% nonunion rate after medial malleolar osteotomy [27]. A decrease in plantarflexion was another complication seen after malleolar osteotomy [28]. We did not encounter nonunion, malunion, or plantar flexion deficiency in our mosaicplasty group of 19 patients. We believe that with the correct technique, medial malleolar osteotomy will not result in any complications.

Robinson et al. and Schumann et al. compared cartilage repair techniques (autologous chondrocyte transplantation or osteochondral autografting) with bone marrow stimulation techniques (microfracture or arthroscopic drilling) and reported similarly positive outcomes for both strategies [12,29]. These authors suggested that despite being associated with biomechanically weaker fibrocartilage formation, bone marrow stimulation techniques should be the first treatment option for osteochondral lesions in the talus. On the other hand, autologous chondrocyte implantation or osteochondral autografting has been recommended for lesions that do not respond to stimulation techniques and require revision. We believe that given the similar outcomes of both treatment methods in our study, the less invasive microfracture technique should be attempted first for talus osteochondral lesions; however, mosaicplasty should be performed in cases where microfracture is not beneficial, revision is required, cyst-based, or for larger lesions.

The study most closely related to ours in the existing literature is the study conducted by Güney et al. Güney and colleagues compared microfracture and mosaicplasty methods, highlighting mosaicplasty as a more effective method for pain compared to microfracture [7]. However, in our study, we found that both methods have similar effects on pain and ankle function. While our study had a relatively short follow-up period of approximately two years, Güney et al. followed their patients for about four years. The different results between the two studies may be attributed to the difference in follow-up periods. Literature suggests that fibrous cartilage is less durable than hyaline cartilage and may weaken after two years [12,29]. This raises the question of whether mosaicplasty should also be performed for lesions smaller than 1.5 cm^2^ if fibrous cartilage weakens after two years. Further studies with longer follow-up periods and larger sample sizes are needed to address this issue.

The limiting factors of our study are the small number of patients (47), short follow-up periods (average of 26 months), and the exclusion of obese and diabetic patients from the study.

## Conclusions

We believe that both treatment methods have similar positive effects on pain and ankle function. The microfracture method offers advantages such as being less invasive and requiring a shorter procedure time. On the other hand, mosaicplasty allows for healing with hyaline cartilage and can be used for larger lesions. However, it is a more invasive method and may cause knee pain. Therefore, microfracture should be the preferred treatment for small osteochondral lesions. We advocate for the application of the microfracture method for lesions smaller than 1.5 cm^2^, while mosaicplasty should be considered for lesions larger than 15 cm^2^ or those not benefiting from microfracture, as well as for cystic lesions. However, larger controlled studies with longer follow-up periods are needed to reach a definitive conclusion.
